# Phylogenetic Analysis of *Scaphoideus* Reveals New Insights Into the Invasion History of *Scaphoideus titanus* (Hemiptera, Cicadellidae) in Europe

**DOI:** 10.1002/ece3.71976

**Published:** 2025-08-20

**Authors:** Juan Sebastian Enciso Garcia, Erika Corretto, Lapo Ragionieri, Luciano Palmieri, Edel Peréz‐López, Christopher Dietrich, Eric Lombaert, Katrin Janik, Hannes Schuler

**Affiliations:** ^1^ Faculty of Agricultural, Environmental and Food Sciences Free University of Bozen‐Bolzano Bozen‐Bolzano Italy; ^2^ Competence Centre for Plant Health Free University of Bozen‐Bolzano Bozen‐Bolzano Italy; ^3^ Département de Phytologie, FSAA; CRIV; IBIS; L'institute EDS Université Laval Québec City Canada; ^4^ Illinois Natural History Survey, Prairie Research Institute University of Illinois at Urbana– Champaign Champaign Illinois USA; ^5^ INRAE, Université Côte d'Azur ISA Sophia Antipolis France; ^6^ Molecular Biology and Microbiology Laimburg Research Centre Pfatten‐Vadena Italy

**Keywords:** cryptic species, mitochondrial genomes, morphological variation, phylogeny, species complex

## Abstract

The genus *Scaphoideus* is one of the most diverse groups in Deltocephalinae with significant pest species, including the American grapevine leafhopper *Scaphoideus titanus* Ball, the vector of Flavescence dorée phytoplasma in European grapevines. Despite the diversity and the agricultural importance of these species, limited information regarding the phylogenetic relationships of *Scaphoideus* species is available. Furthermore, although *S. titanus* is one of the most important pest of grapevine in Europe, details on its intraspecific genetic variation are limited to only a few studies based on single mitochondrial markers. By sequencing the complete mitochondrial genomes of *S. incisus* Osborn, *S. melanotus* Osborn, *S. minor* Osborn, *S. nigrisignus* Li, and *S. titanus*, we examined the phylogenetic relationships among these species. We specifically investigated the genetic differences between *S. titanus* populations from North America and Europe to reveal new insights on the invasion history of this species in Europe. Based on our dataset, two distinct genetic clades of *S. titanus* with more than 10% pairwise genetic distance were identified in the native range: one comprising Midwestern USA and Quebec, Canada and a second one with individuals from Ontario, Canada. Morphological analysis of the aedeagus showed differences between the two clades, suggesting that they should be considered as different species. Invasive European populations share high mitochondrial similarity with those from Ontario, supporting the hypothesis that the invasion originated from this region. Our data reveal new insights into the invasion history of this important pest species and highlight the need for a taxonomic revision of the *S. titanus* species complex.

## Graphical abstract

In this study we investigated the evolutionary relationships of Scapohoideus species and specifically investigated the genetic differences between *Scaphoideus titanus* populations from North America and Europe to reveal new insights on the invasion history of this species in Europe. Sequencing the mitochondrial genomes of several Scaphoideus species, two genetically distinct groups of *S. titanus* in North America, which also differ in morphology and may represent separate species. European populations closely match those from Ontario, Canada, suggesting this region as the invasion source. These findings improve our understanding of *S. titanus* invasion history and indicate a need for taxonomic revision in the species complex.

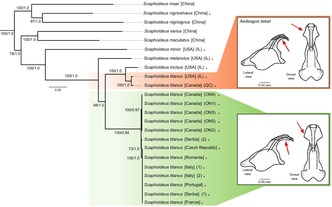

## 1 Introduction

The genus *Scaphoideus* Uhler is one of the largest within the Deltocephalinae subfamily (Hemiptera: Membracoidea) with more than 200 species described in North America, South East Asia, Africa, and Europe (Chen and Dai 2015; Kamitani and Hayashi 2013; Viraktamath and Mohan 2004). Despite the high number of species, to date, phylogenetic reconstructions have been insufficiently discussed on the basis of morphological and molecular characters (Barnett 1977; Du et al. 2017; Hamilton 1983). Barnett (1977) proposed a phylogeny of Nearctic species based on morphological characters, but molecular assessments included only four *Scaphoideus* species from China for which the complete mitochondrial genomes are available (Du et al. 2017). A few additional studies focused only on the mitochondrial genes *cox1* and *cox2* that have limited phylogenetic resolution (Papura et al. 2012; Gonella et al. 2024).

Among the known *Scaphoideus*, two species play a crucial role as vectors transmitting phytoplasmas. In North America, *Scaphoideus luteolus* Van Duzee is the vector of ‘*Candidatus* Phytoplasma ulmi’, which causes elm yellows disease, leading to phloem necrosis in elm trees (Lee et al. 2004). In Europe, the Nearctic leafhopper *Scaphoideus titanus* is the primary vector of the Palearctic '*Candidatus* Phytoplasma vitis' which is causing Flavescence dorée, one of the most important grapevine diseases in Europe (Chuche and Thiéry 2014). The temperate North American species of *Scaphoideus* were revised taxonomically by Barnett (1977), whose concept of *S. titanus* includes five junior synonyms and is distributed in Eastern and Central North America (Barnett 1977; Dmitriev et al. 2022). Hamilton (1983) considered it a species complex and interpreted the species differently than Barnett (1977) removing *S. diutius* DeLong & Mohr and *S. aduncus* DeLong and Knull, adding the synonym *cyprius*. Initial characterization of the mitochondrial gene *cox1* suggested that *S. titanus* as defined by Barnett constitutes multiple genetically distinct lineages (Gonella et al. 2024).

The native distribution of *S. titanus* (*sensu* Barnett) spans most of the regions of deciduous forest in the USA and Southern Canada (Beanland et al. 2006; Olivier et al. 2009; Gonella et al. 2024), especially east of the Rocky Mountains (Barnett 1977). Although *S. titanus* was most likely introduced into Europe during the importation of American vines into France around the 1850s, or during the Phylloxera crisis around 1872 (Gale 2002), the first official record dates to 1958 in Southern France (Bonfils and Schvester 1960). Subsequently, *S. titanus* was found in many European countries, including Italy (Belli et al. 2010; Vidano 1964), Switzerland (Clerc et al. 1997), Portugal (Sousa et al. 2011), Eastern European countries (Mirutenko et al. 2018) and European island territories such as Madeira Island in Portugal (Aguin‐Pombo et al. 2020). However, the geographic source of its introduction and the exact time of its initial arrival remain unclear. Low genetic variation of *S. titanus* in Europe is consistent with the single introduction hypothesis (Bertin et al. 2007; Papura et al. 2012).

In this study, we aimed to infer potential invasion routes of *S. titanus* from North America to Europe by comparing individuals from the native and invasive ranges using morphological and molecular approaches. The presence of two distinct clades among North American specimens led us to further describe the genetic variability between species from the native and invasive ranges. For this purpose, we extended our phylogenetic analyses to other related *Scaphoideus* species from North America ( *S. incisus* , *S. melanotus* , *S. minor* ), one from Asia (*S. nigrisignus*) as well as four previously published species (Du et al. 2017). Our findings offer new insights not only into the invasion history of *S. titanus*, one of the most significant pest species in Europe, but also into additional related species from the native range.

## 2 Materials and Methods

### 2.1 Sample Collection and Morphological Characterization

Five *Scaphoideus* species were analyzed in this study: *S. incisus* , *S. melanotus* , *S. minor* , *S. nigrisignus*, and *S. titanus*. Samples were collected between 2004 and 2023 in China, Canada, USA, and six European countries (Table 1). Identification at the species level was accomplished by examining specimens with a stereomicroscope (Leica S9i) and using the dichotomous key of Barnett (1977) and Wen et al. (2017).

**TABLE 1:** List of complete mitochondrial genomes assembled in this study together with information about the sampling localities and mitochondrial genome length.

Species	Sample ID	Life stage	Sex	Sampling location	Coordinates	Accession Number	Mitochondrial genome length
*S. incisus*	—	Adult	Female	Mason, IL, USA	40°10′22.80″ N 90°54′25.20″ W	PV613518	15,397
*S. melanotus*	—	Adult	Female	Vermillion, IL, USA	40°03′32.40″ N 87°33′54.00″ W	PV613519	15,675
*S. minor*	—	Adult	Female	Pope county, Illinois, USA	37°33′48.6″ N 88°38′32.7″ W	PV613520	14,890
*S. nigrisignus*	—	Adult	Female	Zheijiang, Tianmushan, China	30°05′34.08″ N 118°55′43.32″ E	PV613521	14,675
*S. titanus*	Czech Republic	Adult	Female	Brno, Czech Republic	48°44′24″ N 16°44′28″ E	PV613503	15,619
	France	Adult	Female	Burgundy, France	—	PV613504	15,508
	Italy 1	Nymph	—	Parodi, Italy	44°40′9.94″ N 8°45′13.91″ E	PV613505	15,426
	Italy 2	Adult	Male	Ora, Italy	46°15′44.9″ N 11°11′42.5″ E	PV613506	15,725
	Portugal	Adult	Female	—	—	PV613507	15,425
	Romania	Adult	Female	Odobesti, Romania	44°37′01.3″ N 25°32′36.4″ E	PV613508	14,792
	Serbia 1	Adult	Male	Malca, Serbia	43°20′46.3″ N 22°02′19.4″ E	PV613509	16,171
	Serbia 2	Adult	Female	Belgrade, Serbia	44°51′19.3″ N 20°22′39.1″ E	PV613510	15,538
	Canada, QC	Adult	—	Ile d'Orlean, QC, Canada	46°54′36.8″ N 71°01′28.8″ W	PV613511	15,739
	USA, IL	Adult	Male	Vermillion, IL, USA	40°05′27.0″ N 87°50′11.0″ W	PV613512	15,553
	Canada, ON1	Adult	—	Ontario Peninsula, ON, Canada	43°09′1.7″ N 79°25′0.56″ W	PV613513	15,288
	Canada, ON2	Adult	—	Ontario Peninsula, ON, Canada	43°09′1.7″ N 79°25′0.56″ W	PV613514	15,457
	Canada, ON3	Adult	—	Ontario Peninsula, ON, Canada	43°09′1.7″ N 79°25′0.56″ W	PV613515	15,427
	Canada, ON4	Adult	—	Ontario Peninsula, ON, Canada	43°09′1.7″ N 79°25′0.56″ W	PV613516	15,427
	Canada, ON5	Adult	—	Ontario Peninsula, ON, Canada	43°09′1.7″ N 79°25′0.56″ W	PV613517	15,288

For the morphological analysis of *S. titanus*, 24 male specimens (four from France, three from Serbia 1, two from Italy 2, two from IL, USA, and 13 from QC, Canada) were dissected. The male genitalia were examined with a focus on variations in size, length, and shape of the major structures to assess the consistency of these morphological features across specimens from the native and invasive range. After taxonomic identification, all specimens were stored directly in absolute ethanol at −20°C prior to DNA extraction.

### 2.2 DNA Extraction and Illumina Sequencing

A total of 19 individuals were analyzed in this study. DNA from five individuals from the Ontario Peninsula, ON, Canada was obtained from a previously published study (Papura et al. 2012). Additionally, DNA from 14 individuals from the five *Scaphoideus* species (one individual from *S. incisus* , *S. melanotus* , *S. minor, S. nigrisignus* and ten individuals from *S. titanus*; Table 1) was extracted using the DNeasy blood & tissue extraction kit according to the manufacturer's instructions (Qiagen, Hilden, Germany). DNA quality and quantity were measured using the DS‐11 FX+ (Denovix Inc., Wilmington, DE, USA) fluorometer and the Qubit 1× dsDNA high sensitivity (HS) kit (Invitrogen, Waltham, DE, USA). Paired‐end 2 × 150 bp Illumina Novaseq sequencing was performed at Macrogen Europe (Amsterdam, The Netherlands).

### 2.3 Mitochondrial Genome Assembly, Annotation and Analysis

Raw reads were mapped against the mitochondrial genome of *S. maai* (KY817243) using BWA V0.7.17‐r1188 (Li and Durbin 2009) to select only reads belonging to the mitochondrial genome. Mapped reads were assembled using SPAdes v3.15.4 with default parameters (Bankevich et al. 2012). The assembly produced a single contig representing the complete circular mitochondrial genome for each specimen, except for *S. minor* , for which the mitochondrial genome was split into two contigs. To close the gap observed between the two contigs of *S. minor* , we designed new primers: SMgapF 5′—CTCAGACCTTCATGTTTTAA—3′ and SMgapR 5′—GCCTAGGGTTAGTTTCATAT—3′. PCR was performed in a total volume of 25 μL containing 2 μL of DNA, 1.75 μL of each primer (10 μM), 12.5 μL of 2X DreamTaq Master Mix (ThermoFisher) and 7 μL of sterile water under the following thermal conditions: 3 min at 95°C for initial denaturation; 35 cycles of 30 s at 95°C, 30 s at 48°C, 1 min at 72°C, and 10 min at 72°C. PCR products were sent for Sanger sequencing at Eurofins Genomics (Ebersberg, Germany). Annotation of the mitochondrial genomes was performed in MITOS2 v2.1.3 (Bernt et al. 2013) and then manually curated. The newly assembled mitogenomes were deposited in the NCBI under accession numbers PV613503–PV613521.

The non‐synonymous (Ka) and synonymous (Ks) substitution rates of each protein coding gene (PCG) together with the Ka/Ks ratio were calculated in DnaSP v6.12.03 (Rozas et al. 2017) using the 19 genomes assembled in this study and the ones published by Du et al. (2017). The Ka/Ks dataset was tested for normality of variance using the Shapiro–Wilk test in R v4.3.1 using the packages FSA and ggsignif. The Kruskal–Wallis rank‐sum test was conducted to analyze the differences of Ka/Ks ratios across the PCGs in *Scaphoideus*, and a multiple comparison test was conducted using Bonferroni correction to compare the gene evolutionary rates.

## 3 Phylogenetic Analysis

The phylogenetic analyses were performed using the 19 newly assembled mitochondrial genomes (Table 1) along with *S. maai* (KY817243), *S. maculatus* (NC_060770), *S. nigrivalveus* (KY817244) and *S. varius* (KY817245). Two Deltocephalinae species, *Roxasellana stellata* (NC_050257) and *Drabescoides nuchalis* (NC_028154) served as outgroups. Alignments of the nucleotide sequences of the 13 PCGs were generated with the MAFFT online server with Auto option algorithm (Katoh et al. 2019). The final alignments were concatenated with FASconCAT‐G (Kück and Longo 2014). Maximum likelihood estimation (ML) was performed in IQTREE v2.3.6 (Minh et al. 2020). IQTREE was run on the concatenated matrix with the MFP + MERGE option using ModelFinder (Kalyaanamoorthy et al. 2017) applying an Edge‐unlinked partition model. Node support was assessed using 1000 ultrafast bootstrap replicates (Hoang et al. 2018). Additionally, Bayesian inference (BI) analysis was performed using MrBayes v3.2 (Ronquist et al. 2012). The analysis was conducted with two independent runs, each consisting of four Markov chains, run for 50 million generations with sampling every 5000 generations. The GTR + I + G substitution model was applied, as determined by ModelFinder. A burn‐in of 25% of the sampled trees was applied before summarizing the posterior distribution. Convergence was assessed by ensuring the average standard deviation of split frequencies fell below 0.01. A stop rule and checkpointing were enabled to monitor convergence and ensure data recovery in case of interruption. Posterior probabilities were calculated from the majority‐rule consensus tree. Trees were visualized using FigTree (http://tree.bio.ed.ac.uk/software/figtree/). Finally, a haplotype network was constructed using the concatenated alignment of the 13 PCGs of all *S. titanus* individuals with the Templeton, Crandall & Sing (TCS) algorithm as implemented in PopArt (http://popart.otago.ac.nz).

## 4 Results

### 4.1 Analysis of the Mitochondrial Genomes of Five *Scaphoideus* Species

The size of the 19 complete mitochondrial genomes of the five *Scaphoideus* species ranged from 14,890 bp in *S. minor* to 16,171 bp in *S. titanus* from France and Serbia 1 (Table 1). The newly assembled circular mitochondrial genomes contained a complete set of 13 PCGs, two rRNAs, and 22 tRNAs. Gene order was conserved across all mitogenomes, with *atp*6, *atp*8, *cytB*, *cox*1, *cox*2, *cox*3, *nad*3, and *nad*6 located on the J‐strand, and *nad*1, *nad*4, *nad4*l, and *nad*5 on the N‐strand.

The average overall ratio of non‐synonymous and synonymous substitutions (Ka/Ks) was positive for all the 13 PCGs analyzed in *Scaphoideus* species. The analysis of synonymous (Ks) and non‐synonymous (Ka) substitution rates in the 13 PCGs revealed that *nad4* has the lowest Ka/Ks value, while *nad2* has the highest (Figure S1). The results indicate that *atp8*, *cox1, cox2, nad1, and nad2* are under positive selection (Ka/Ks > 1), whereas *atp6*, *cytB, cox3, nad3, nad4, nad4l*, *nad5*, and *nad6* are under purifying selection (Ka/Ks < 1).

## 5 Morphological analysis of *Scaphoideus titanus*

**FIGURE 1:**
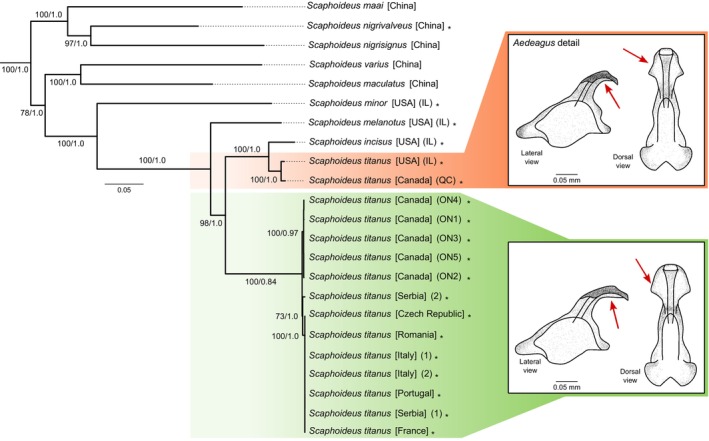
Phylogenetic tree based on the concatenated nucleotide sequences of the 13 mitochondrial protein‐coding genes (PCGs) from *Scaphoideus* species. Numbers on branches indicate node support values from Maximum Likelihood (ML) and Bayesian Inference (BI) analyses, shown as ML bootstrap/BI posterior probability. Terminal labels marked with an asterisk (*) indicate newly assembled mitochondrial genomes generated in this study. Morphological detail of the two aedeagus variants in *S. titanus*, shown in lateral and dorsal views in the inset figures. Arrows indicate the observed differences in the apical part of the aedeagus. Outgroups were removed to improve visualization.

We dissected and compared the male genitalia of *S. titanus* to assess morphological congruency across populations from the native (IL, USA; ON and QC, Canada) and invasive (France, Italy, Serbia) range (Figure 1). The number, size, and position of setae on the pygofer exhibited noticeable variation among the specimens studied; though this variation was observed not only between but also within populations and was not deemed taxonomically significant. As the long setae can easily fall off from the pygofer, we counted the setal bases, which proved to be more consistent across populations.

The internal genital structures, including the aedeagus, connective, and styles, were generally consistent between European and North American populations. The length and shape of the process of the connective displayed no variation among the observed specimens. However, a noticeable variation was observed in individuals from IL, USA, and QC, Canada, where the apical part of the aedeagus was shorter and had a less prominent “shield” when compared to the European specimens and individuals from ON, Canada (Figure 1). No other morphological divergence was observed between the males from IL, USA, and QC, Canada, and individuals from other populations of *S. titanus*.

## 6 Phylogenetic Relationships Within *Scaphoideus*

The phylogenetic analyses demonstrated strong bootstrap support for the majority of nodes within the genus *Scaphoideus*. However, the clade comprising *S. varius* and *S. maculatus* exhibited moderate support values, with a ML bootstrap value of 78 and a BI posterior probability of 1.0. *S. nigrisignus* was identified as a sister species to *S. nigrivalveus*, with *S. maai* placed as their sister group. Within the New World clade, *S. minor* and *S. melanotus* formed a strongly supported sister group to the clade containing *S. incisus* and *S. titanus*.

All individuals morphologically identified as *S. titanus* were divided into two strongly supported clades. The first clade included specimens from QC, Canada, and IL, USA, which grouped with *S. incisus* (ML = 100; BI = 1.0). The second clade comprised *S. titanus* from ON, Canada, and from Europe, which also formed subclades (ML ≥ 98; BI = 1.0, except for one internal node within the ON group). Sample Serbia 2 was placed as sister to the remaining European lineage, with strong support (ML = 100; BI = 0.84). The phylogenetic structure within *S. titanus* corresponds with the two observed aedeagus morphotypes, suggesting congruence between molecular and morphological variation (Figure 1).

## 7 Haplotype Network Analysis

**FIGURE 2:**
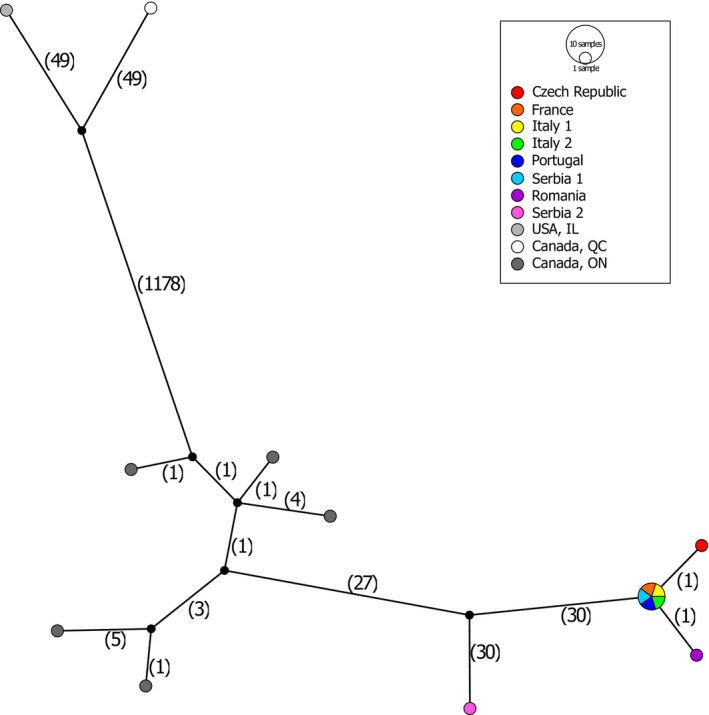
Haplotype (TCS) network for 13 mitochondrial protein coding genes in *Scaphoideus titanus* showing the relationship between populations from the native and invasive range. Numbers of single nucleotide polymorphisms are reported in brackets near the representing nodes.

To visualize the relationships between the different haplotypes, a network was constructed using only samples that were morphologically identified as *S. titanus*. The haplotype network analysis of the 15 mitochondrial genomes of *S. titanus* revealed 12 different haplotypes. Consistent with the phylogenetic reconstruction, two haplotype groups were identified, separated by 1178 substitutions (Figure 2). The first group contained the samples from IL, USA, and QC, Canada, which were separated by 98 mutations. In the second, all remaining populations from ON, Canada, and Europe were included. The five samples from ON Canada, were directly connected to the samples IL, USA, and QC, Canada, differing from each other by one to five mutations. The sample from Serbia 2 was separated from ON, Canada, samples by 27 plus 30 mutations, and from the rest of Europe by 30 plus 30 fixed mutations. These results suggest a possible second source population for these samples, possibly from Southern Canada or North Eastern United States. Additionally, all samples from France, Portugal, Italy, Romania, Czech Republic, and Serbia 1 were genetically homogeneous.

## 8 Discussion

To estimate the phylogenetic relationships between Palearctic and Nearctic *Scaphoideus* species, we inferred the genetic and evolutionary relationships among nine *Scaphoideus* species, with a particular focus on *S. titanus* to examine its invasion history in Europe. We compared the morphology and the mitochondrial genome data from *S. titanus* of 11 localities from the native range in North America and the invasive range in Europe. Our findings reveal new insights into the evolutionary history of the genus *Scaphoideus* and shed new light on the invasion history of *S. titanus* in Europe.

### 8.1 *Scaphoideus* Phylogeny

The mitochondrial genomes of *Scaphoideus* were consistent in structure and organization with most other reported insect and Cicadellidae mitochondrial genomes (Du et al. 2017). Genome length, gene order, and codon usage were highly conserved. However, length variation among *S. titanus* from different localities may be explained by differences in the control region. This non‐coding part of the mitochondrial genome helps regulate DNA replication and gene expression and in hemipterans usually contains short A and T stretches (Li and Liang 2018). The Ka/Ks ratio quantifies selection pressure on a gene, where Ka/Ks = 1 indicates neutral selection, Ka/Ks < 1 indicates purifying selection, and Ka/Ks > 1 indicates positive selection (Hurst 2002). Our Ka/Ks analysis showed that nine out of 13 PCGs are under positive selection, while three are under purifying selection. Interestingly, high rates in Ka/Ks are rare in Deltocephalinae species (Wang et al. 2015) but common in Sternorrhyncha, likely due to mitochondrial rearrangements (Liu et al. 2020; Hou et al. 2023). The genes *cox1* and *cox2* are largely evolving under positive selection with ratios greater than 1 (Figure S1). Even if they are currently both widely used as mitochondrial markers, alternative genes, such as *nad2* and *atp8*, may provide additional resolution.

Our results show the clade comprising all included New World *Scaphoideus* derived within a paraphyletic grade comprising the two clades of included East Asian species, suggesting that the genus originated in the Old World and subsequently colonized North America. Newly sequenced *S. nigrisignus* from China was placed as sister to *S. nigrivalveus*, which shares common phenotypical features such as the absence of pale longitudinal stripes in dorsal view and the crown with a complete transverse band (Wen et al. 2017). Within the New World clade, *S. minor* and *S. melanotus* grouped together as sister clade to the remaining *Scaphoideus* from the Nearctic region, differing from traditional taxonomy, which clustered *S. melanotus* and *S. titanus* as closely related taxa (Barnett 1977).

### 8.2 Two Highly Divergent Clades of *S. titanus*

Our phylogenetic analysis revealed two distinct clades of *S. titanus*, one including individuals from the Midwestern USA and QC, Canada and another from ON, Canada and Europe. These two clades also exhibit morphological differences in the aedeagus. The concept of *S. titanus* adopted by Barnett (1977) was broad, encompassing several previously described species that were treated as synonyms. Prior to our study, analysis of the mitochondrial gene *cox1* of *S. titanus* specimens also recovered two separate clades (Gonella et al. 2024). However, no morphological differences were assessed, nor was genetic differentiation examined until the present study.

*Scaphoideus titanus* as described in Barnett (1977) exhibited considerable variation, with overlap in many morphological characteristics with the nearest species as *S. cyprius* and *S. nigrellus*. Differences in the paraphyses length, size, and shape allowed morphological identification in males. The aedeagus was described as highly variable but generally pear‐shaped, with structural differences best observed in the posterior view. Barnett (1977) recognized four distinct morphotypes of the aedeagus within *S. titanus*, a level of variation not observed in any of the other 20 *Scaphoideus* species treated in the same revision. In our study, we found differences in the apical part of the aedeagus between three populations of North America, which are also reflected by molecular analyses. In the native range, *S. titanus* is more diverse than in the invasive range, where it is described as polyphagous, infesting multiple plants (Barnett 1977). This study supports those observations, noting, for example, that *S. titanus* specimens from QC, Canada, were captured in strawberry fields not surrounded by *Vitis vinifera* (Plante et al. 2024). In contrast, in Europe, this species is described to be monophagous, feeding almost exclusively on *V. vinifera* and occasionally in other species of *Vitis* (Chuche and Thiéry 2014).

The two morphotypes observed in this study are consistent with two of the four morphological variants illustrated by Barnett (1977). Few individuals from IL, USA and QC, Canada were included in our morphological and molecular analysis. Therefore, further investigations with a larger number of specimens will be needed to determine whether individuals from IL, USA and QC, Canada included in our study represent different species. However, analyses of the complete mitochondrial genomes have proven to be a powerful approach for phylogenetics and population genomics (Shao and Barker 2007; Molligan et al. 2024). Our results indicate it is possible that *S. titanus* comprises multiple allopatric species occurring in different areas and/or co‐occurring in the same areas. Therefore, hybridization including mitochondrial introgression may take place, and a single locus approach may be insufficient to detect hybridization. Based on the available data, the most likely scenario for the origin of European populations of *S. titanus* is a single introduction from founding populations from Ontario or the Northeastern USA. However, secondary introductions may have occurred, as suggested by genetically divergent individuals from Serbia 2, potentially originating from a different source population, possibly still from this range. Future studies are needed to investigate the whole *S. titanus* species complex described by Barnett (1977) and the geographic distribution of these species in North America to understand the diversity and evolution of *S. titanus* in the native range.

## 9 Conclusion

Here we sequenced the complete mitochondrial genomes of *S. incisus* , *S. melanotus* , *S. minor* , *S. nigrisignus*, and *S. titanus* for the first time and analyzed these data in combination with previously sequenced mitochondrial genomes from other *Scaphoideus* species, with emphasis on the invasion history and morphology of *S. titanus* across North America and Europe. Our study reveals striking genetic and morphological differences between populations of *S. titanus*, suggesting that these clades may represent distinct species. While additional taxonomic work is needed to test this hypothesis, particularly using larger morphological datasets and genomic tools, our results highlight the need to revisit the species boundaries of one of the most important viticulture pests in its native range. Especially, the fact that these cryptic species might possess the potential to become new invasive pests and should be considered in monitoring programs.

## Author Contributions

**Juan Sebastian Enciso Garcia:** conceptualization (equal), data curation (lead), formal analysis (lead), methodology (lead), software (equal), visualization (equal), writing – original draft (equal), writing – review and editing (equal). **Erika Corretto:** conceptualization (equal), data curation (lead), formal analysis (lead), methodology (lead), software (lead), visualization (equal), writing – original draft (lead), writing – review and editing (equal). **Lapo Ragionieri:** conceptualization (equal), methodology (equal), visualization (equal), writing – review and editing (supporting). **Luciano Palmieri:** conceptualization (equal), data curation (equal), methodology (equal), visualization (equal), writing – review and editing (equal). **Edel Peréz‐López:** validation (equal), writing – review and editing (equal). **Christopher Dietrich:** visualization (equal), writing – review and editing (equal). **Eric Lombaert:** resources (equal), writing – review and editing (equal). **Katrin Janik:** conceptualization (supporting), funding acquisition (equal), resources (equal), writing – review and editing (equal). **Hannes Schuler:** conceptualization (lead), funding acquisition (lead), project administration (lead), resources (equal), supervision (lead), visualization (equal), writing – original draft (equal), writing – review and editing (equal).

## Conflicts of Interest

The authors declare no conflicts of interest.

## Supporting information


**Figure S1:** Boxplots of the Ka/Ks (Ka, non‐synonymous substitution; Ks, synonymous substitution) ratio of the 13 mitochondrial protein coding genes shared by all *Scaphoideus* species. The estimates were based on pairwise alignments of samples. Asterisks above the boxplots indicate significant differences (***p < 0.001) between the Ka/Ks ratios of the coding genes based on Dunn's test with Bonferroni correction.

## Data Availability

The mitochondrial genomes assembled in this study are available as Supporting Information and will be openly available at the NCBI database under the accession numbers PV613503–PV613521.
