# Supplementary material for: Phylogenetic Analysis of Scaphoideus Reveals New Insights Into the Invasion History of Scaphoideus titanus (Hemiptera, Cicadellidae) in Europe
Source: Ecol Evol. 2025 Aug 20;15(8):e71976. doi: 10.1002/ece3.71976 (PMC12367863; doi:10.1002/ece3.71976)
Supplement: Supplementary file 1 — Figure S1: Boxplots of the Ka/Ks (Ka, non‐synonymous substitution; Ks, synonymous substitution) ratio of the 13 mitochondrial protein coding genes shared by all Scaphoideus species. The estimates were based on pairwise alignments of samples. Asterisks above the boxplots indicate significant differences (***p < 0.001) between the Ka/Ks ratios of the coding genes based on Dunn's test with Bonferroni correction. [file ECE3-15-e71976-s001.docx]

**
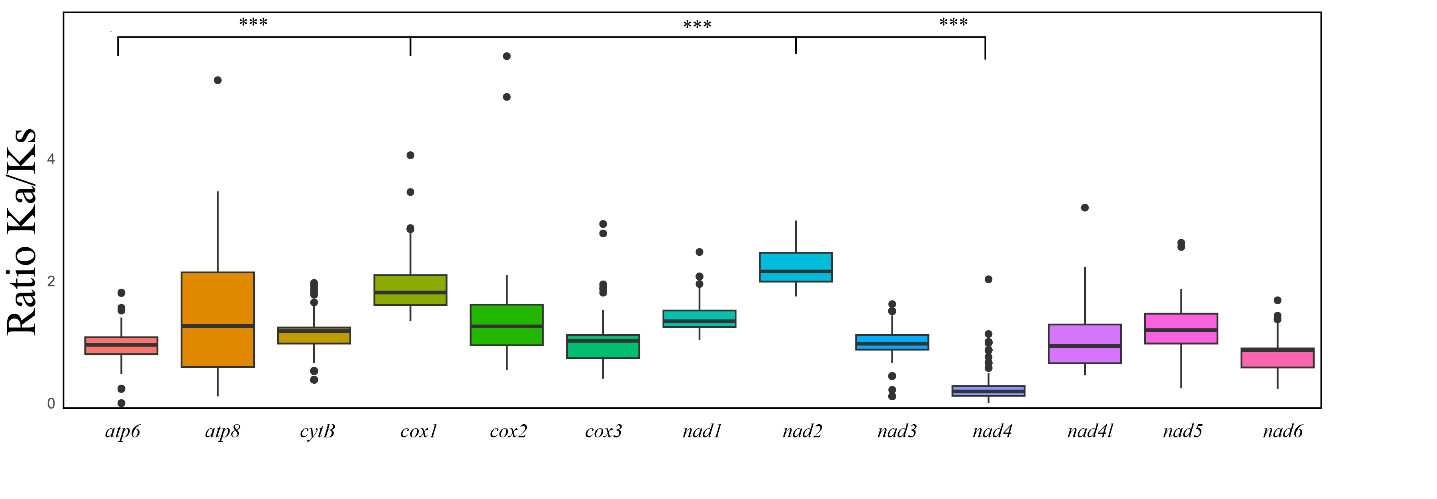
**

**Figure S1.** Boxplots of the Ka/Ks (Ka, non-synonymous substitution; Ks, synonymous substitution) ratio of the 13 mitochondrial protein coding genes shared by all *Scaphoideus* species*.* The estimates were based on pairwise alignments of samples. Asterisks above the boxplots indicate significant differences (***P<0.001) between the Ka/Ks ratios of the coding genes based on Dunn´s test with Bonferroni correction.
